# Dynamic Alterations of the Gut Microbial Pyrimidine and Purine Metabolism in the Development of Liver Cirrhosis

**DOI:** 10.3389/fmolb.2021.811399

**Published:** 2022-01-28

**Authors:** Yinghui Xiong, Li Wu, Li Shao, Yang Wang, Zebing Huang, Xun Huang, Chunhui Li, Anhua Wu, Zhenguo Liu, Xuegong Fan, Pengcheng Zhou

**Affiliations:** ^1^ The Hunan Provincial Key Laboratory of Viral Hepatitis, Department of Infectious Diseases, Xiangya Hospital, Central South University, Changsha, China; ^2^ Department of Infectious Diseases, Infection Control Center, The Third Xiangya Hospital, Central South University, Changsha, China; ^3^ Institute of Translational Medicine, The Affiliated Hospital of Hangzhou Normal University, Hangzhou, China; ^4^ Institute of Integrative Medicine, Department of Integrated Traditional Chinese and Western Medicine, Xiangya Hospital, Central South University, Changsha, China; ^5^ Infection Control Center, Xiangya Hospital, Central South University, Changsha, China

**Keywords:** liver cirrhosis, gut microbiota, pyrimidine and purine metabolism, fecal metabolomics, metagenomics sequencing

## Abstract

**Background:** Liver cirrhosis is the common end-stage of liver disease which lacks effective treatment, thus studies to determine prevention targets are an urgent need. The intestinal microbiota (IM) play important roles in modulating liver diseases which are mediated by microbial metabolites. Despite decades of growing microbial studies, whether IM contribute to the development of cirrhosis and the intimate metabolic link remain obscure. Here, we aimed to reveal the dynamic alterations of microbial composition and metabolic signatures in carbon tetrachloride (CCl_4_)-induced liver cirrhosis mice.

**Methods:** CCl_4_-treated mice or normal control (NC) were sacrificed (*n* = 10 per group) after 5 and 15 weeks of intervention. The disease severity was confirmed by Masson’s trichrome or Sirius red staining. Metagenomics sequencing and fecal untargeted metabolomics were performed to evaluate the composition and metabolic function of IM in parallel with the development of cirrhosis.

**Results:** The CCl_4_-treated mice presented liver fibrosis at 5 weeks and liver cirrhosis at 15 weeks indicated by collagen deposition and pseudo-lobule formation, respectively. Mice with liver cirrhosis showed distinct microbial composition from NC, even in the earlier fibrosis stage. Importantly, both of the liver fibrosis and cirrhosis mice were characterized with the depletion of Deltaproteobacteria (*p* < 0.05) and enrichment of Akkermansia (*p* < 0.05). Furthermore, fecal metabolomics revealed distinguished metabolomics profiles of mice with liver fibrosis and cirrhosis from the NC. Notably, pathway enrichment analysis pointed to remarkable disturbance of purine (*p* < 0.001 at 5 weeks, *p* = 0.034 at 15 weeks) and pyrimidine metabolic pathways (*p* = 0.005 at 5 weeks, *p* = 0.006 at 15 weeks) during the development of liver cirrhosis. Interestingly, the disorders of pyrimidine and purine metabolites like the known microbial metabolites thymidine and 2′-deoxyuridine had already occurred in liver fibrosis and continued in cirrhosis.

**Conclusion:** These novel findings indicated the crucial role of IM-modulated pyrimidine and purine metabolites in the development of liver cirrhosis, which provides microbial targets for disease prevention.

## 1 Introduction

Liver cirrhosis is prevalent globally, contributing to 1 million annual deaths worldwide (Ginès et al., 2021). Cirrhosis results from chronic liver inflammation followed by diffuse liver fibrosis and eventually develops into liver failure (Pellicoro et al., 2014). Currently, the management of liver cirrhosis is primarily focused on treating the causes and complications (Ginès et al., 2021). The reversible therapeutics of cirrhosis remain an unconquered area, and its progress is hampered due to the unclear mechanism underlying the disease development (Schuppan and Kim, 2013).

During the past decades, studies have indicated the potential role of gut microbiota in the pathogenesis of liver cirrhosis. Researchers have found the alterations of gut microbiota in patients with liver cirrhosis (Qin et al., 2014; Bajaj et al., 2021) and revealed the association between gut dysbiosis and its complications and poorer prognosis including liver failure and hepatocellular carcinoma (Bajaj and Khoruts, 2020; Moreno-Gonzalez and Beraza, 2021; Trebicka and Bork, 2021). Despite the increase of studies, gut microbiota was reported to involve in the development of liver cirrhosis, but which kind of microbiota and its role are still obscure.

Mechanistic studies presented the role of bacterial translocation in the progression of liver cirrhosis, while the increase of bacteria permeation was absent in the early stage of liver cirrhosis (Wiest et al., 2014). These findings suggested that there were messengers mediating the communication between gut microbiota and liver during the development of liver cirrhosis. The gut microbiota were considered as an “organ” producing a large array of metabolites which facilitated the crosstalk between gut microbiota and the host (Nicholson et al., 2012). Thus, the fecal metabolomics study which provided a functional readout of gut microbiota (Zierer and Jackson, 2018) might reveal the metabolic link underlying the interactions between gut microbiota and liver during the development of liver cirrhosis.

In order to identify the microbial signatures driving the development of liver cirrhosis, we integrated metagenomics sequencing and fecal untargeted metabolomics techniques in a carbon tetrachloride (CCl_4_)-induced liver cirrhosis mice model at different time points. These dynamic findings in the current study would help in elucidating the metabolic mechanism of gut microbiota in the development of liver cirrhosis and provide a microbial target for disease prevention.

## 2 Methods and Materials

### 2.1 Animal Experiment

The animal experiments were reviewed and approved by the Animal Ethics Committee of Central South University (Permit number: 2019sydw0056) and conducted according to the Guide for Animal Care and Use. Male C57BL/6J mice (8 weeks old, 18–20 g) were purchased from SLAC Laboratory (Hunan, China). All mice were housed in one cage for 1 week of acclimation, thus ensuring a similar microbial composition at baseline. Then mice were randomly divided into two groups (*n* = 20 per group): 1) the CCl_4_ group, mice were intraperitoneally (i. p.) injected with CCl_4_ solution (Macklin Biochemical, China) (20% in olive oil, 3 ml/kg body weight, twice a week); 2) the normal control (NC) group, mice were i. p. injected with an equal volume of olive oil (Macklin Biochemical, China). All mice were maintained in a temperature and light controlled specific pathogen-free facility (23°C, 12 h dark-light cycle, and 50% humidity) with free access to feed and water. To observe the evolution of gut microbiota in parallel with the development of cirrhosis, mice in the NC and CCl_4_ groups were sacrificed at different time points (5 and 15 weeks after intervention, *n* = 10 per group at each time point).

### 2.2 Measurement of Liver Function Parameters

Blood samples were obtained and centrifuged (3,000 rpm, 15 min) to collect serum. The serum levels of aminotransferase (ALT), aspartate aminotransferase (AST), alkaline phosphatase (ALP), gamma-glutamyl transferase (γ-GT), total bilirubin (TBIL), and total bile acid (TBA) in mice were detected by an automated chemistry analyzer, Chemray 240 (Rayto Life Science, China).

### 2.3 Histological Evaluation of the Liver

After fixing in formalin, liver tissues were embedded in paraffin. In order to assess liver injury, the 3-μm-thick liver sections were stained with hematoxylin and eosin (H&E). Additionally, the tissues were stained with Masson’s trichrome or Sirius red, and morphometric analysis was performed. Briefly, five randomly selected 40 × fields were analyzed by the ImageJ software (NIH, United States) (Syn et al., 2012).

### 2.4 Analysis of Microbial Community by Metagenomics Sequencing

The feces of mice were freshly collected at the 5th and 15th week of the intervention and immediately stored at −80°C until analysis. The total DNA was extracted from feces samples of mice, and the DNA quality and concentration was evaluated by 1% agarose gel and a Qubit® dsDNA Assay Kit (Life Technologies, CA, United States), respectively. Then, the DNA samples were fragmented and prepared for PCR amplification. Next, the PCR products were purified and used for library construction. And the library preparations were sequenced using an Illumina HiSeq platform.

The acquired raw data were preprocessed by Readfq to obtain clean data which were further assembled and analyzed using SOAPdenovo software (Luo et al., 2012), predicted by MetaGeneMark (Qin et al., 2014), and annotated by DIAMOND software (Gao et al., 2021). The α diversity indexes reflecting microbial richness (Chao1) and diversity (Shannon index) were calculated by the R vegan package. The PERMANOVA was used to evaluate β-diversity [principal coordinates analysis (PCoA)] based on Canberra distance. The linear discriminant analysis (LDA) effect size (LEfSe) was performed to identify the differential taxa between groups, and the microbiota with *p* values <0.05 and LDA scores >2 were considered statistically significant.

### 2.5 Fecal Metabolomics Analysis

#### 2.5.1 Chemicals

HPLC-grade acetonitrile was purchased from Merck (Germany), and ammonium acetate was purchased from Sigma (United States). Ammonia and methanol were purchased from Thermo Fisher Scientific (United States).

#### 2.5.2 Sample Preparation

The fecal samples were first vortexed with water, and then metabolites were extracted by mixing with solvent (methanol/acetonitrile (v:v) = 1:1) in the ratio of 1:4. Next, the mixtures were centrifuged at 14,000 g for 20 min and the supernatants were obtained. Equal volume of the extracted samples were mixed as quality control (QC) samples to ensure the quality of metabolic profiling.

#### 2.5.3 Untargeted Metabolomics Profiling

The fecal metabolites were analyzed with the Agilent 1,290 Infinity ultra high performance liquid chromatography (UHPLC) platform (Agilent, United States). An ACQUITY UPLC BEH Amide column (2.1 × 100 mm, 1.7 µm, Waters, United States) was used for compound separation at 25°C. The eluents employed in both electrospray ionization positive (ESI +) and negative models (ESI −) were A (water containing 25 mM ammonium acetate and 25 mM ammonia) and B (acetonitrile). The elution gradient was set as 40–95% of the gradient of B. The mass spectral data were acquired by AB Triple TOF 6600 mass spectrometry (AB SCIEX, United States) in both ESI + and ESI − mode. And the MS-MS data were collected by information-dependent acquisition.

#### 2.5.4 Data Analysis

The raw data were processed using XCMS software (Ma et al., 2020) for peak alignment, retention time adjustment, and peak extraction. And compounds identification was performed by referring to an in-house database (Shanghai Applied Protein Technology Co., Ltd). Then, partial least squares discriminant analysis (PLS-DA) of multivariate data was conducted in SIMCA-P 13.0 (Umetrics AB, Sweden). And the differential metabolites were identified with variable importance in projection (VIP) values more than 1 and univariate analysis showed *p* values less than 0.1 between groups (Wu et al., 2021). Next, hierarchical cluster analysis of differential metabolites was performed using Cluster 3.0 software (Wu et al., 2021) and enriched pathway analysis was conducted in Metaboanalyst (v.4.0) (Páez-Franco et al., 2021).

### 2.6 Statistical Analysis

Data were given as means with standard deviation (mean ± SD), or medians with ranges. Statistical analysis was performed in SPSS software package version 22.0 (SPSS Inc., United States) and GraphPad Prism 6 (GraphPad Software, United States). The Student’s t-test or Mann-Whitney U test was used for group comparison, and *p* value <0.05 was considered as a significant difference.

## 3 Results

### 3.1 CCl_4_ Gradually Induced Liver Cirrhosis in Mice

**FIGURE 1:**
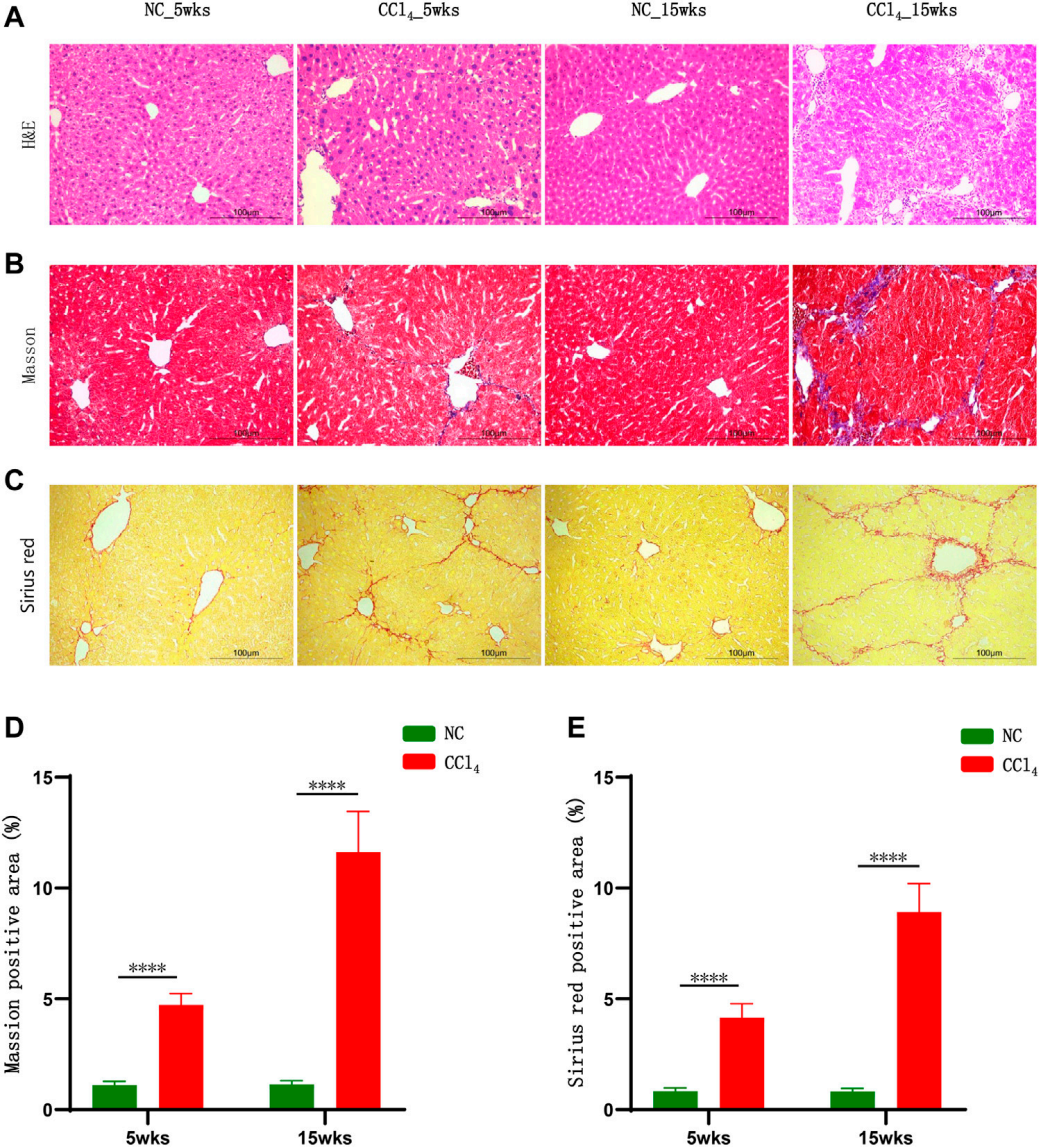
CCl_4_ gradually induced liver cirrhosis in mice. **(A–C)** Representative H&E, Masson’s trichrome, and Sirius red staining of liver in mice after 5 and 15 weeks of CCl_4_ treatment. **(D, E)** Morphometric analysis of Masson’s trichrome and Sirius red staining; results were presented as the percentage of positive area. Note: Data were given as mean ± SD. n: **(D, E)** 10 per group. ****p < 0.0001. Abbreviations: NC, normal control; CCl_4_, carbon tetrachloride; H&E, hematoxylin and eosin; SD, standard deviation.

As shown in Figure 1A, the H&E staining results showed that 5-weeks CCl_4_ intervention caused liver injury indicated by the structural disorder of the hepatic cord, swollen hepatocytes, and mild inflammatory cell infiltration without obvious hepatocyte necrosis. Although the serum parameters reflecting hepatocytes injury including ALT, AST, and TBIL were unchanged (Supplementary Figures S1A–C), the serum ALP indicating cholangitis was significantly increased in mice with 5-weeks CCl_4_ compared with those with vehicles (Supplementary Figure S1E). And the serum TBA and γ-GT were also unchanged in CCl_4_-treated mice (Supplementary Figures S1D,F). Following liver injury, the Masson’s trichrome and Sirius red staining results presented collagen deposition in the portal area which was extended into the hepatic lobule (Figures 1B,C), and the morphometric analysis results showed that the percentage of the positive area of Masson’s trichrome and Sirius red staining was significantly higher in CCl_4_-treated mice than NC after 5 weeks of intervention (Figures 1D,E). These results showed that 5-weeks CCl_4_ intervention induced liver fibrosis in mice.

The 15-weeks CCl_4_ administration resulted in more severe liver injury indicated by a stretched and deformed bile duct, moderate inflammatory cell infiltration, and hepatocytes enlargement shown by H&E staining (Figure 1A). Meanwhile, the serum cholangitis markers ALP and γ-GT were increased and the serum levels of TBIL and TBA were higher in CCl_4_-treated mice compared with NC (Supplementary Figures S1C–F). Similar to findings after 5 weeks, the ALT and AST levels were unchanged (Supplementary Figures S1A,B) and there was no obvious hepatocyte necrosis (Figure 1A). Parallel with the progression of liver injury, Masson’s trichrome and Sirius red staining exhibited marked fibrogenesis and pseudo-lobule formation in mice with 15-weeks CCl_4_ treatment (Figures 1B,C). And the positive staining area of Masson’s trichrome and Sirius red staining was much higher in CCl_4_-treated mice than NC (Figures 1D,E). These results evidenced that mice with 15-weeks CCl_4_ administration developed liver cirrhosis without necrosis, which was consistent with previous findings that showed that CCl_4_ may induce liver cirrhosis by causing bile duct injury and abnormal bile excretion rather than hepatocyte necrosis (Horikoshi et al., 2015; Li et al., 2018).

### 3.2 Dynamic Alterations of Gut Microbiota During the Development of Liver Cirrhosis in Mice

**FIGURE 2:**
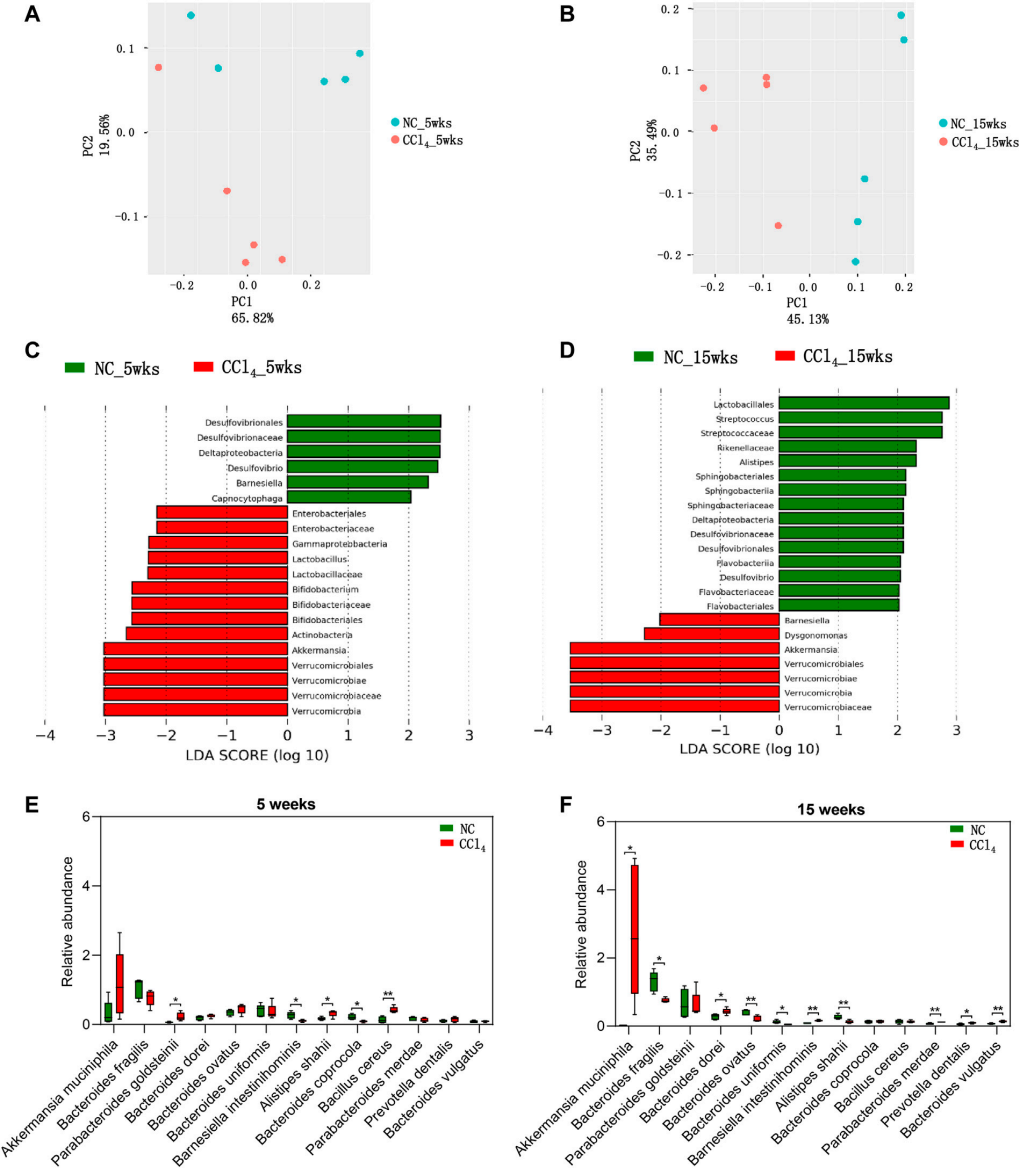
Dynamic alterations of microbial composition in mice during the development of CCl_4_-induced liver cirrhosis. **(A, B)** The β-diversity of gut microbiota is shown by PCoA plots based on Bray-Curtis dissimilarity. **(C, D)** LEfSe analysis was performed using the predominant microbiota from phylum, class, order, family, and genus levels. The cladogram represents enriched taxon in the NC group (green) and CCl_4_ treatment groups (red) with an LDA score >2 at 5 weeks or 15 weeks after intervention. **(E, F)** The predominant species were compared between NC and CCl_4_-treated mice after 5-weeks or 15-weeks intervention. Note: Data were given as medians with range. n: **(A–F)** 5 per group. *p < 0.05; **p < 0.01. Abbreviations: NC, normal control; CCl_4_, carbon tetrachloride; PCoA, principal co-ordinates analysis; LEfSe, Linear discriminant analysis Effect Size; LDA, linear discriminant analysis.

In order to truly reveal the microbial alterations in the development of liver cirrhosis, metagenomic sequencing was performed. There was no significant difference between CCl_4_-treated mice and NC in Chao l and Shannon indexes after 5 and 15-weeks intervention (Supplementary Figures S2A,B), indicating that microbial richness and diversity were unchanged during the development of liver cirrhosis. However, as shown in Figures 2A,B, the PCoA plots displayed the distinct microbial composition between mice with 5 or 15-weeks CCl_4_ treatment (PERMANOVA, *p* = 0.021 and 0.011, respectively). Furthermore, LEfSe analysis based on microbes from phylum, class, order, family, and genus was conducted to identify the microbiota associated with the development of liver cirrhosis. As shown in Figure 2C, mice with 5-weeks CCl_4_ showed an increase of microbiota from Verrucomicrobia including *Verrucomicrobiae*, *Verrucomicrobiales*, *Verrucomicrobiaceae,* and *Akkermansia* compared with those with vehicles. Meanwhile, the gut microbiota from Actinobacteria including *Bifidobacteriales*, *Bifidobacteriaceae,* and *Bifidobacterium*, and *Lactobacillaceae* as well as its genera *Lactobacillus* were also significantly increased in CCl_4_-treated mice. The class *Gamaproteobacteria* as well as *Enterobacteriales* and its common pathogens *Enterobacteriaceae* were enriched in mice with 5-weeks CCl_4_ intervention. Reversely, the microbes of *Deltaproteobacteria* including *Desulfovibrionales*, *Desulfovibrionaceae,* and *Desulfovibrio* were decreased in the CCl_4_-treated group compared to NC*.* The genera *Barnesiella* and *Capnocytophaga* were also reduced in mice with 5-weeks CCl_4_ treatment. These results showed altered microbial composition in the liver fibrosis mice induced by 5-weeks CCl_4_.

For the liver cirrhosis mice induced by 15-weeks CCl_4_, gut microbiota from *Lactobacillales* including *Streptococcaceae* and *Streptococcus* were decreased compared with NC. Formicrobes from Bacteroidetes and *Dysgonomonas* were increased while taxa of *Sphingobacteriia* (*Sphingobacteriales*, *Sphingobacteriaceae*), *Flavobacteriia* (*Flavobacteriales*, *Flavobacteriaceae*), and *Rikenellaceae* and its genera *Alistipes* were decreased in mice of the CCl_4_ group compared to NC. Unlike the findings of 5-weeks intervention, *Barnesiella* was increased in CCl_4_-treated mice. Notably, consistent with the trend in liver fibrosis mice, liver cirrhosis mice also presented with enriched microbiota from Verrucomicrobia including *Verrucomicrobiae*, *Verrucomicrobiales*, *Verrucomicrobiaceae,* and *Akkermansia,* and the depletion of *Deltaproteobacteria*as well as its order *Desulfovibrionales*, family *Desulfovibrionaceae,* and genus *Desulfovibrio* (Figure 2D). These findings support the vital role of gut microbiota, especially Verrucomicrobia and *Deltaproteobacteria*, in the development of liver cirrhosis.

Due to the distinct microbiota from phylum to genus levels during the development of liver cirrhosis, we further analyzed the microbes at species level. Among all of the predominant species with the average prevalence of more than 0.1% in any groups, 13 species were significantly altered in mice with 5-weeks or 15-weeks CCl_4_ treatment when compared with NC. As shown in Figures 2E,F, four species including *Akkermansia muciniphila*, *Bacteroides dorei*, *Prevotella dentalis*, and *Bacteroides vulgatus* were progressively increased while two species including *Bacteroides fragilis* and *Bacteroides uniformis* were progressively decreased during the development of CCl_4_-induced liver cirrhosis, and showed a significant difference between CCl_4_-treated mice and NC after 15-weeks intervention, indicating modulation of these six species might prevent liver cirrhosis. The significant increase of *Parabacteroides goldsteinii,* and *Bacillus cereus,* and decrease of *Bacteroides coprocola* only presented in mice with liver fibrosis. While the liver cirrhosis mice were featured with increased *Parabacteroides merdae* and decreased *Bacteroides ovatus*. And *Barnesiella intestinihominis* and *Alistipes shahii* showed an opposite alteration trend in liver fibrosis and liver cirrhosis mice when compared with NC. These findings further suggest that gut dysbiosis is involved in the development of liver cirrhosis, suggesting microbial intervention might be a potential target for preventing and treating liver cirrhosis.

### 3.3 Microbial Metabolic Disorders Related to the Development of Liver Cirrhosis

**FIGURE 3:**
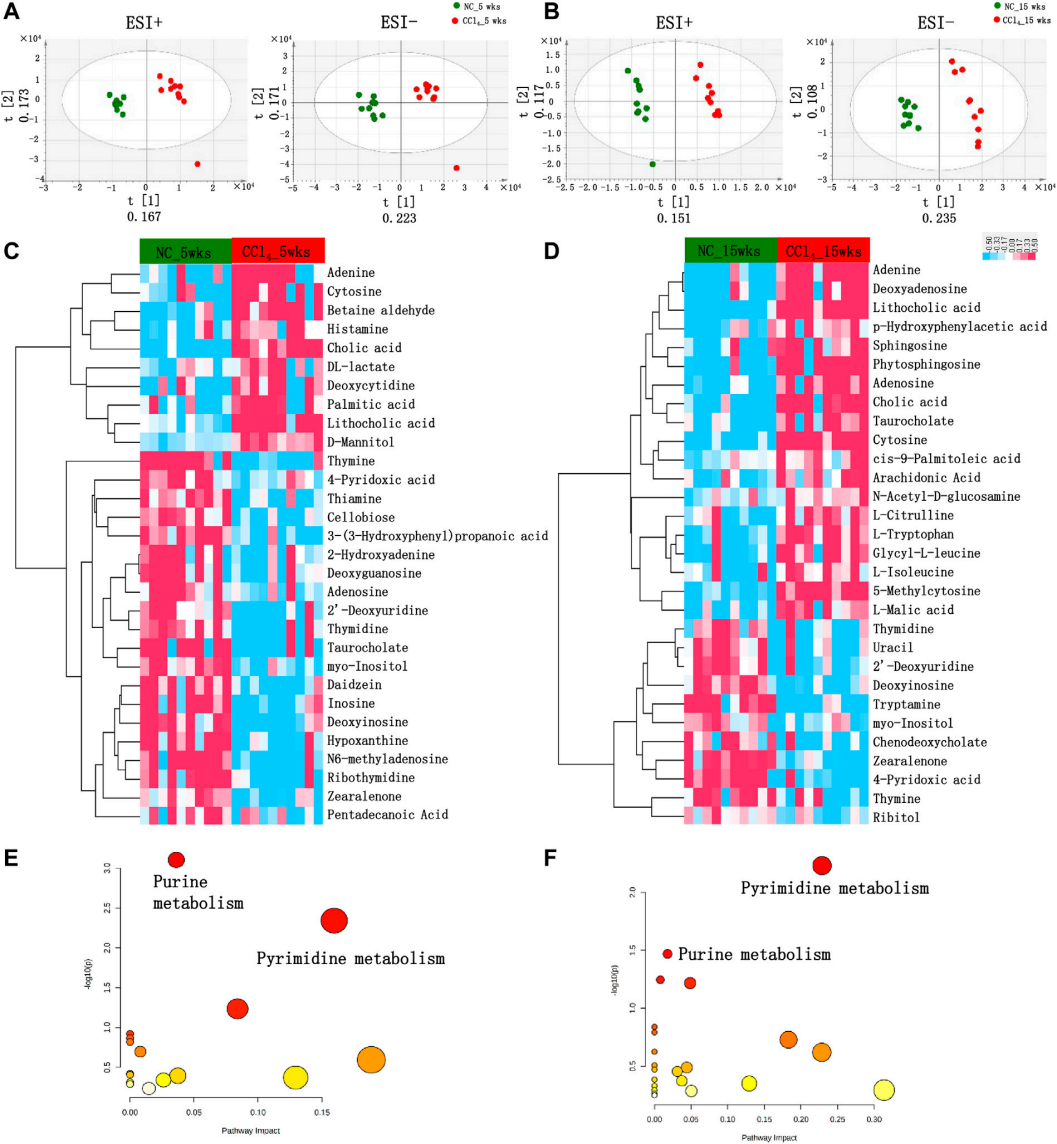
Differential fecal metabolomics profiling in mice during the development of CCl_4_-induced liver cirrhosis. **(A, B)** PLS-DA score plots based on the fecal metabolomics profiles in both ESI+ and ESI− models using UPLC-MS/MS data after 5 or 15-weeks intervention. **(C, D)** The clustering heat map based on the top 15 fecal differential metabolites in both positive and negative modes of mice with 5 or 15-weeks CCl_4_. The colors from blue to red indicate the relative concentration of metabolites in each sample. **(E, F)** The scatter plot shows the pathway enrichment of differential metabolites of mice with 5 or 15-weeks CCl_4_ by referring to KEGG in MetaboAnalyst v.4.0. Note: n: **(A–D)** 10 per group. Abbreviations: NC, normal control; CCl_4_, carbon tetrachloride; PLS-DA, partial least squares discriminant analysis; UPLC-MS, ultraperformance liquid chromatography-tandem mass spectrometry; KEGG, Kyoto Encyclopedia of Genes and Genomes; ESI+, electrospray ionization positive; ESI−, electrospray ionization negative; UPLC-MS/MS, ultra performance liquid chromatography-tandem mass spectrometry.

The compositional alterations of gut microbiota prompted us to further analyze the metabolic disorders of microbial community by fecal metabolomics, which provided a functional readout of microbiota. As shown in Figures 3A,B, the PLS-DA plots based on untargeted fecal metabolomics data of both positive and negative modes showed distinct metabolomics profiles of mice with liver fibrosis and liver cirrhosis from NC. The fecal metabolites with the top 15 VIP values of both positive and negative modes were chose as the differential metabolites involved in the development of liver cirrhosis (Figures 3C,D).

As shown in Figure 3C; Supplementary Table S1, among the 30 differential metabolites in liver fibrosis mice with 5-weeks CCl_4_ intervention, 8 metabolites belong to purine metabolism and 7 were metabolites of pyrimidine metabolism. Among these 15 purine and pyrimidine metabolites, 3 metabolites including adenine, cytosine, and deoxycytidine were enriched in mice with liver fibrosis, while 7 purine metabolites (deoxyinosine, 2-hydroxyadenine, inosine, hypoxanthine, N6-methyladenosine, adenosine, and deoxyguanosine), and 5 pyrimidine metabolites (2′-deoxyuridine, thiamine, thymine, thymidine, and ribothymidine) were decreased in mice with CCl_4_-treatment compared with those with vehicles. Four organic compounds [3-(3-Hydroxyphenyl)propanoic acid, daidzein, cellobiose, and zearalenone] were decreased, and D-mannitol was increased in mice with CCl_4_ compared with NC. The primary bile acid cholic acid and the secondary bile acid lithocholic acid were increased while the conjugated taurocholate was decreased in CCl_4_-treated mice. The lipid metabolites of palmitic acid and pentadecanoic acid were also significantly altered in CCl_4_-treated mice. The CCl_4_ treatment also disturbed the metabolism of pyruvate, vitamin B6, betaine, histidine, and inositol, indicated by altered DL-lactate, 4-pyridoxic acid, betaine aldehyde, histamine, and myo-Inositol compared with NC. The pathway enrichment analysis using these 30 metabolites showed that liver fibrosis mice were characterized by remarkable disturbance of purine (*p* < 0.001) and pyrimidine metabolism (*p* = 0.005) (Figure 3E).

As shown in Figure 3D and Supplementary Table S2, consistent with the liver fibrosis mice, the liver cirrhosis mice induced by 15-weeks CCl_4_ treatment also showed obvious alterations in purine and pyrimidine metabolites, and pathway enrichment also pointed out the dysregulation of purine (*p* = 0.034), and pyrimidine (*p* = 0.006) metabolic pathways (Figure 3F). Similar to the trend in the liver fibrosis mice, cytosine and adenine were increased, and 2′-deoxyuridine, thymidine, thymine, and deoxyinosine were decreased in mice with liver cirrhosis compared with NC. Meanwhile, the enrichment of cholic acid, lithocholic acid, and the depletion of zearalenone, myo-Inositol, and 4-pyridoxic acid were also shown in mice with 15 weeks of CCl_4_ treatment. Unlike the trend at 5 weeks, adenosine and taurocholate were increased in mice with 15-weeks CCl_4_ treatment. Besides, 5-methylcytosine of pyrimidine metabolites and deoxyadenosine of purine metabolites were increased, while uracil of pyrimidine metabolites, and the primary bile acid chenodeoxycholate were decreased in mice with 15-weeks CCl_4_ compared with those with vehicles. Four lipid metabolites including arachidonic acid, phytosphingosine, cis-9-palmitoleic acid, and sphingosine were increased in CCl_4_-treated mice. The mice with 15-weeks CCl_4_ treatment also showed the disorders of amino acid metabolism indicated by the increased N-acetyl-D-glucosamine, L-citrulline, L-tryptophan, *p*-hydroxyphenylacetic acid, L-isoleucine, Glycyl-L-leucine, and decreased tryptamine. The ribitol of organic compounds and L-malic acid of pyruvate metabolism were also obviously altered in feces of mice with 15-weeks CCl_4_ treatment. Taken together, these metabolomics findings revealed the distinct metabolic function of gut microbiota during the development of liver cirrhosis. Notably, the differential metabolites mainly pointed to the disorders of purine and pyrimidine metabolism.

### 3.4 Disturbed Pyrimidine and Purine Metabolism During the Development of Liver Cirrhosis

**FIGURE 4:**
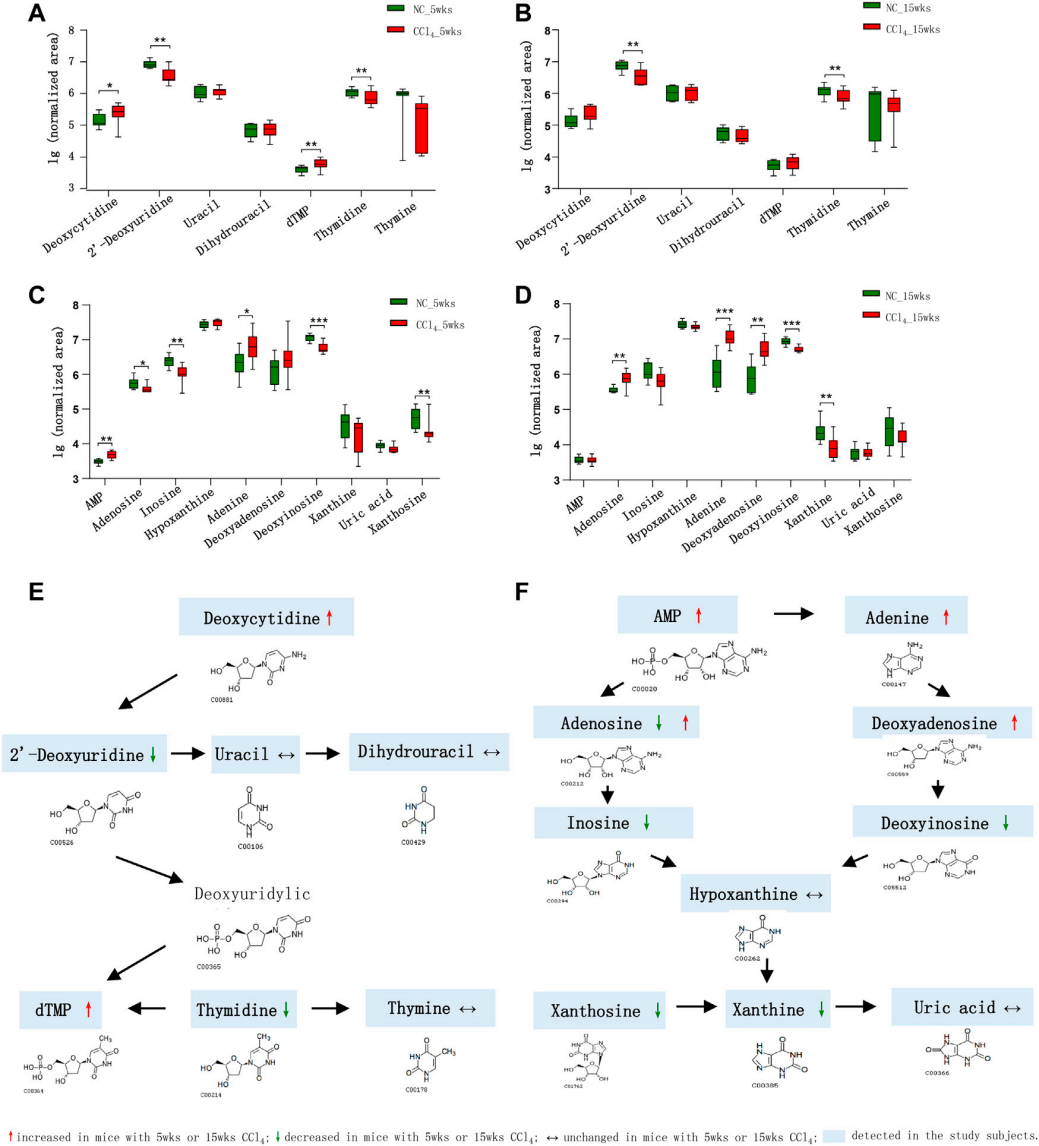
Dysregulation of fecal pyrimidine and purine metabolic pathways in mice during the development of CCl_4_-induced liver cirrhosis. **(A, B)** Comparison of the detected pyrimidine metabolites in the feces of mice with or without CCl_4_ intervention for 5 or 15 weeks. **(C, D)** Comparison of the detected purine metabolites in the feces of mice with or without CCl_4_ intervention for 5 or 15 weeks. **(E, F)** Schematic diagram of the disturbed pyrimidine and purine metabolic pathways in CCl_4_-treated mice. Note: Data were given as median with range. *p < 0.05; **p < 0.01; ***p < 0.001. Abbreviations: NC, normal control; CCl_4_, carbon tetrachloride.

Due to the obvious disturbance of purine and pyrimidine metabolism, we compared all detected metabolites involved in purine and pyrimidine metabolism pathways. As shown in Figures 4A,B,E, in total seven pyrimidine metabolites were detected in feces of all mice. For two pyrimidine deoxyribonucleosides, deoxycytidine was significantly increased while the 2′-deoxyuridine was decreased in mice with 5-weeks CCl_4_-induced liver fibrosis. The thymidine of pyrimidine nucleosides, a known microbial metabolite was decreased, while its downstream dTMP was increased in liver fibrosis mice, indicating the enhanced microbial conversion from thymidine to dTMP. Consistently, the depletion of 2′-deoxyuridine and thymidine was also presented in the liver cirrhosis mice with 15-weeks CCl_4_ treatment. And the nucleobase uracil, thymine, and uracil derivative dihydrouracil were comparable between CCl_4_-treated mice and NC after 5 or 15 weeks of intervention.

In terms of compounds related to purine metabolism, 10 metabolites were identified in our study (Figures 4C,D,F). The nucleotide AMP was significantly increased and its nucleoside adenosine was decreased in mice with liver fibrosis compared with NC. The inosine which could be deaminated from adenosine was also decreased in liver fibrosis mice. While the adenosine was increased in mice with liver cirrhosis. The adenine is the purine base of nucleoside deoxyadenosine, which could be deaminated into deoxyinosine. The mice with liver fibrosis showed the increase of adenine and decrease of deoxyinosine in feces. Interestingly, the liver cirrhosis mice exhibited increased adenine and deoxyadenosine, and decreased deoxyinosine, indicating the enhanced synthesis, and blocked degradation of deoxyadenosine in microbial community during the development of liver cirrhosis. The purine nucleoside xanthosine, also a microbial metabolite, as well as its base xanthine were decreased in mice with liver fibrosis or liver cirrhosis compared with NC. The hypoxanthine and the end product of purine metabolism uric acid were unchanged in CCl_4_-treated mice. These findings supported the fact that the disturbance of pyrimidine and purine metabolism was involved in the development of liver fibrosis.

## 4 Discussion

In the current study, we revealed the dynamic alterations of gut microbiota in the development of liver cirrhosis and found that *Deltaproteobacteria* was decreased while Verrucomicrobia was increased in the earlier liver fibrosis and the later liver cirrhosis mice. Furthermore, the untargeted fecal metabolomics results reflecting the functional readout of gut microbiota showed that the predominant metabolic disturbance of microbiota pointed to the pyrimidine and purine metabolism. Interestingly, these metabolic disorders already occurred in liver fibrosis and persisted in liver cirrhosis. Our novel findings suggested the crucial role of gut microbiota in the pathogenesis of liver cirrhosis, and firstly noted the pyrimidine and purine metabolites as important mediators of “gut-liver” axis in promoting disease development.

Mice injected with 5-weeks CCl_4_ developed liver fibrosis and developed into liver cirrhosis at 15 weeks indicated by the pseudolobuli formation. The microbes from *Deltaproteobacteria* such as *Desulfovibrionaceae* were significantly decreased in both liver fibrosis and cirrhosis mice. Plenty of studies reported the adverse role of *Deltaproteobacteria* in intestinal tissue due to its sulfate-reducing bacteria with the capacity of producing the toxic H_2_S (Fox et al., 1994; Van Hecke et al., 2019). Actually, bacteria in *Deltaproteobacteria,* including the genus *Desulfovibrio* containing superoxide dismutase, and has been reported to play important roles in protecting against the detrimental effects of superoxide free radicals (Bruschi et al., 1977). Similar to our findings, the decrease of *Desulfovibrionaceae* was also reported in nonalcoholic fatty liver disease (Yun et al., 2019). And *Deltaproteobacteria* was recommended as a probiotic to control pathogenic lung bacteria forcystic fibrosis (CF) in patients (de Dios Caballero et al., 2017). Therefore, the depletion of *Deltaproteobacteria* might contribute to the development of liver cirrhosis and the ecological significance requires further study.

Inversely, Verrucomicrobia as well as its genera *Akkermansia* and species *Akkermansia muciniphila* were increased since the onset of liver fibrosis. Consistent with our findings, the enriched *Akkermansia* was also reported in patients with cirrhosis when compared with HCC-cirrhosis patients (Lapidot et al., 2020). And the increase of *Akkermansia* was also presented in primary sclerosing cholangitis patients (Kummen et al., 2017) and bile duct ligation mice (Cabrera-Rubio et al., 2019), which were characterized by fibrosis. *Akkermansia* was the only representative microbe of the phylum Verrucomicrobia in the host gut and extensively existed in the intestinal mucosa to use the mucin as the sole source of carbon and nitrogen elements (Zhang et al., 2019; Xu et al., 2020). However, the controversial role of mucin in different diseases might determine the diverse effect of *Akkermansia* (Cabrera-Rubio et al., 2019; Xie and Fei, 2021). For example, *Akkermansia muciniphila* was recommended as the promising probiotic against metabolic disorders (Zhang et al., 2019), while the studies in a high-fructose (Wang et al., 2020) or high-fat diet showed the increase of *Akkermansia* (Xie and Fei, 2021), and *Akkermansia* could reduce the important enzymes which prevented hyperglycemia and hyperlipidemia (Chleilat et al., 2021). Besides, the enrichment of *Akkermansia* has been reported in inflammatory states such as alcohol-induced steatohepatitis (Ran et al., 2021). Here, we revealed the increase of *Akkermansia* related to the development of liver cirrhosis, suggesting modulation on *Akkermansia* might prevent liver cirrhosis.

The changing trend of fecal metabolites in our findings is similar to that found in patients with cirrhosis (Wei et al., 2016; Wei et al., 2018; Yang et al., 2018), implying the association of fecal metabolite alteration with disease development, and not the role of CCl_4_. In the present study, the untargeted fecal metabolomics results revealed that mice with liver fibrosis and cirrhosis were characterized with disturbance of pyrimidine and purine metabolism. Several studies indicated the modulating role of gut microbiota in the metabolism of pyrimidine and purine. For example, the enriched *Bacteroides dorei* in our mice with liver cirrhosis contained the thymidine kinase genes involved in thymidine metabolism (Sakanaka et al., 2018). And studies in germ-free mice demonstrated that gut microbiota modulated the metabolism of adenosine (Mager and Burkhard, 2020) and thymidine (Kim et al., 2021). The gut microbiota-derived purine metabolites including eATP and xanthine were related to the development of inflammatory bowel disease (Scott and Gutiérrez-Vázquez, 2021) and high-fat diet-induced obesity (Wei et al., 2021), respectively. And the microbial metabolized inosine of adenosine metabolites mediated the immunotherapy responses of cancer (Allen-Vercoe and Coburn, 2020). To our knowledge, these findings firstly noted the pyrimidine and purine metabolites represented as the metabolic messengers of gut microbiota relating to liver cirrhosis.

The alteration of pyrimidine metabolites including the depletion of 2′-deoxyuridine and thymidine were presented in both liver fibrosis and liver cirrhosis mice. Consistently, the pyrimidine metabolism disorders related to liver diseases like acute-on-chronic liver failure (Zaccherini et al., 2021) and the removal efficacy dysfunction of 2′-deoxyuridine was reported in patients with hepatitis C virus (Czarny et al., 2017). Thymidine was the nucleotide containing a pyrimidine or purine base which was unique to DNA and was widely used for measuring the rate of cell proliferation (Bading and Shields, 2008). Therefore, we speculated that the gut microbiota-modulated thymidine depletion in the present study might suppress the proliferation of liver parenchymal cells after injury, thus promoting fibrogenesis and eventually resulting in liver cirrhosis. These findings suggested that targeting the decreased pyrimidine metabolites has potential for preventing liver cirrhosis.

The concentration of extracellular nucleotides remains low under physiological conditions and turns out to be high in pathological status (Alvarez et al., 2021), indicating that the persistent increase of purine metabolites like adenine in our model mice might be the key event in the development of liver cirrhosis. Consistent with our findings in the feces, Hernández-Muñoz *et al.* revealed the altered adenine nucleotides in the liver of mice with early and late liver fibrosis (Hernández-Muñoz et al., 1990). Adenine was a key purine nucleobase in nucleic acids, and its derivatives regulated various physiological processes such as energy metabolism (Frenguelli and Dale, 2020), tumor microenvironment (Alvarez et al., 2021), brain injury (Frenguelli and Dale, 2020), and insulin secretion of β cells and adipogenesis (Nicholls, 2016). Moreover, the purine metabolites also severed as extracellular signaling molecules to modulate matrix deposition during fibrosis (Ferrari et al., 2016). Inhibiting the receptor of purine metabolites attenuated collagen formation and the activation of hepatic stellate cells in the mice with liver fibrosis (Huang et al., 2014). We found that the adenosine of purine metabolites was significantly decreased in the mice with liver fibrosis but increased in liver cirrhosis, which might be attributed to the diverse roles of adenosine in fibrosis process as reported previously (Hernández-Muñoz et al., 1997; Scott and Gutiérrez-Vázquez, 2021). Generally, the integrated findings of metagenomics and fecal metabolomics in our study implicated the crucial role of microbiota-derived purine metabolites in the development of liver cirrhosis.

## 5 Conclusion

In summary, the current study revealed the compositional and functional alterations of gut microbiota during the development of liver cirrhosis. Our findings showed that liver cirrhosis was characterized with the depletion of *Deltaproteobacteria* and enrichment of *Akkermansia* even in the earlier liver fibrosis stage. Notably, the untargeted metabolomics pointed to the dysregulation of pyrimidine and purine metabolism in the microbial community. These novel findings indicated the vital role of gut microbiota in the development of liver cirrhosis during which pyrimidine and purine metabolites acted as key microbial mediators. However, the precise mechanism of “gut microbiota-pyrimidine or purine metabolites-liver axes” in liver cirrhosis requires further systematic studies. Altogether, our results provided evidence that modulating gut microbiota as well as its pyrimidine and purine metabolites hold promise for preventing liver cirrhosis.

## Data Availability

The datasets presented in this study can be found in online repositories. The names of the repository/repositories and accession number(s) can be found below: https://www.ncbi.nlm.nih.gov/, PRJNA773919.
