# Supplementary material for: Dynamic Alterations of the Gut Microbial Pyrimidine and Purine Metabolism in the Development of Liver Cirrhosis
Source: Front Mol Biosci. 2022 Jan 28;8:811399. doi: 10.3389/fmolb.2021.811399 (PMC8832137; doi:10.3389/fmolb.2021.811399)
Supplement: Supplementary file 1 [file DataSheet1.pdf]

1. Supplementary Tables

Supplementary Table 1. Differential fecal metabolites between mice with 5-week CCl_4 and NC group.

VIP	RT (s)	m/z	Adduct	Identified results	Related pathway	Fold change (CCl_4/NC)	*P*-value
20.86	183.14	815.57	(2M-H)-	Cholic acid	Bile acid metabolism	9.81	0.009
18.87	152.43	136.06	(M+H)+	Adenine	Purine Metabolism	3.45	0.040
12.13	175.36	251.08	(M-H)-	Deoxyinosine	Purine Metabolism	0.51	0.000
11.85	112.18	227.07	(M-H)-	2'-Deoxyuridine	Pyrimidine Metabolism	0.47	0.001
9.74	187.51	112.05	(M+H)+	Cytosine	Pyrimidine Metabolism	3.09	0.003
8.40	350.47	265.11	M+	Thiamine	Pyrimidine Metabolism	0.57	0.001
7.89	45.15	255.23	(M-H)-	Palmitic acid	Lipids metabolism	2.37	0.023
7.69	149.26	165.05	(M-H)-	3-(3-Hydroxyphenyl)propanoic acid	Organic compounds metabolism	0.37	0.002
6.95	219.88	152.06	(M+H)+	2-Hydroxyadenine	Purine Metabolism	0.60	0.082
6.74	45.85	241.22	(M-H)-	Pentadecanoic Acid	Lipids metabolism	0.75	0.043
6.21	100.21	127.05	(M+H)+	Thymine	Pyrimidine Metabolism	0.31	0.000
5.53	44.38	255.06	(M+H)+	Daidzein	Organic compounds metabolism	0.32	0.001
5.51	220.21	89.02	(M-H)-	DL-lactate	Pyruvate Metabolism	1.39	0.079
5.23	208.89	267.07	(M-H)-	Inosine	Purine Metabolism	0.47	0.001

5.08	83.78	241.08	(M-H)-	Thymidine	Pyrimidine Metabolism	0.59	0.029
5.02	41.85	184.06	(M+H)+	4-Pyridoxic acid	Vitamin B6 metabolism	0.65	0.007
4.60	46.78	375.29	(M-H)-	Lithocholic acid	Bile acid metabolism	2.76	0.000
4.40	294.60	162.11	$(M+CH_3COO+2H)+$	Betaine aldehyde	Betaine Metabolism	15.27	0.032
4.31	124.97	137.05	(M+H)+	Hypoxanthine	Purine Metabolism	0.38	0.029
4.26	396.87	360.15	$(M+NH_4)+$	Cellobiose	Organic compounds metabolism	0.59	0.000
3.60	138.89	257.08	(M-H)-	Ribothymidine	Pyrimidine Metabolism	0.33	0.002
3.20	245.30	514.28	(M-H)-	Taurocholate	Bile acid metabolism	0.12	0.010
3.10	40.81	317.14	(M-H)-	Zearalenone	Organic compounds metabolism	0.57	0.001
3.09	129.07	282.12	(M+H)+	N6-methyladenosine	Purine Metabolism	0.22	0.003
3.04	163.92	268.10	(M+H)+	Adenosine	Purine Metabolism	0.67	0.028
2.87	307.94	163.06	$(M-H_2O-H)-$	D-Mannitol	Organic compounds metabolism	1.57	0.000
2.84	382.22	112.09	(M+H)+	Histamine	Histidine Metabolism	2.88	0.056
2.70	381.04	179.06	(M-H)-	myo-Inositol	Inositol Metabolism	0.49	0.002
2.65	221.03	268.10	(M+H)+	Deoxyguanosine	Purine Metabolism	0.55	0.093
2.59	199.16	455.19	(2M+H)+	Deoxycytidine	Pyrimidine Metabolism	1.81	0.027

Abbreviations: VIP, variable importance in projection; RT, retention time; CCl_4, carbon tetrachloride; NC, normal control.

Supplementary Table 2. Differential fecal metabolites between mice with 15-week CCl_4 and NC group.

VIP	RT (s)	m/z	Adduct	Identified results	Related pathway	Fold change (CCl_4/NC)	*P*-value
28.83	152.43	136.06	(M+H)+	Adenine	Purine metabolism	6.59	0.000
21.28	183.14	815.57	(2M-H)-	Cholic acid	Bile acid metabolism	10.26	0.000
18.61	138.04	252.11	(M+H)+	Deoxyadenosine	Purine metabolism	5.21	0.001
13.36	187.51	112.05	(M+H)+	Cytosine	Pyrimidine metabolism	6.83	0.000
9.34	42.25	303.23	(M-H)-	Arachidonic Acid	Lipid metabolism	1.56	0.004
8.74	249.46	222.10	(M+H)+	N-Acetyl-D-glucosamine	Amino sugar metabolism	1.34	0.035
8.57	175.36	251.08	(M-H)-	Deoxyinosine	Purine metabolism	0.59	0.000
8.52	112.18	227.07	(M-H)-	2'-Deoxyuridine	Pyrimidine metabolism	0.53	0.004
6.34	377.91	176.10	(M+H)+	L-Citrulline	Arginine and Proline metabolism	1.40	0.073
5.86	163.92	268.10	(M+H)+	Adenosine	Purine metabolism	2.29	0.002
5.42	247.51	205.10	(M+H)+	L-Tryptophan	Tryptophan metabolism	2.10	0.004
5.39	81.82	243.10	(M+H)+	Thymidine	Pyrimidine metabolism	0.66	0.044
5.24	265.03	130.09	(M-H)-	L-Isoleucine	Valine, Leucine and Isoleucine Degradation	1.55	0.044
5.14	153.25	514.28	(M-H)-	Taurocholate	Bile acid metabolism	2.88	0.009
5.01	46.78	375.29	(M-H)-	Lithocholic acid	Bile acid metabolism	3.85	0.000

4.40	49.89	127.05	(M+H)+	Thymine	Pyrimidine metabolism	0.46	0.036
4.20	110.57	113.03	(M+H)+	Uracil	Pyrimidine metabolism	0.57	0.012
3.92	116.12	375.29	(M+H-H_2O)+	Chenodeoxycholate	Bile acid metabolism	0.55	0.022
3.86	184.41	161.11	(M+H)+	Tryptamine	Tryptophan metabolism	0.14	0.010
3.27	395.97	133.01	(M-H)-	L-Malic acid	Pyruvate metabolism	1.81	0.000
3.18	104.81	300.29	(M+H)+	Sphingosine	Lipid metabolism	1.82	0.017
3.16	181.95	126.07	(M+H)+	5-Methylcytosine	Pyrimidine metabolism	3.10	0.000
2.96	89.41	317.14	(M-H)-	Zearalenone	Organic compounds metabolism	0.52	0.000
2.94	125.65	300.29	(M+H-H_2O)+	Phytosphingosine	Lipid metabolism	3.47	0.000
2.62	289.65	161.04	(M-H_2O-H)-	myo-Inositol	Inositol metabolism	0.75	0.025
2.39	45.13	253.22	(M-H)-	cis-9-Palmitoleic acid	Lipid metabolism	1.37	0.007
2.12	274.32	133.05	(M-H_2O-H)-	Ribitol	Organic compounds metabolism	0.78	0.009
2.06	273.32	187.11	(M-H)-	Glycyl-L-leucine	Valine, Leucine and Isoleucine Degradation	2.12	0.011
1.76	191.38	151.04	(M-H)-	p-Hydroxyphenylacetic acid	Tyrosine Metabolism	1.60	0.009
1.75	104.64	182.04	(M-H)-	4-Pyridoxic acid	Vitamin B6 metabolism	0.40	0.001

Abbreviations: VIP, variable importance in projection; RT, retention time; CCl_4, carbon tetrachloride; NC, normal control.

2. Supplementary Figures

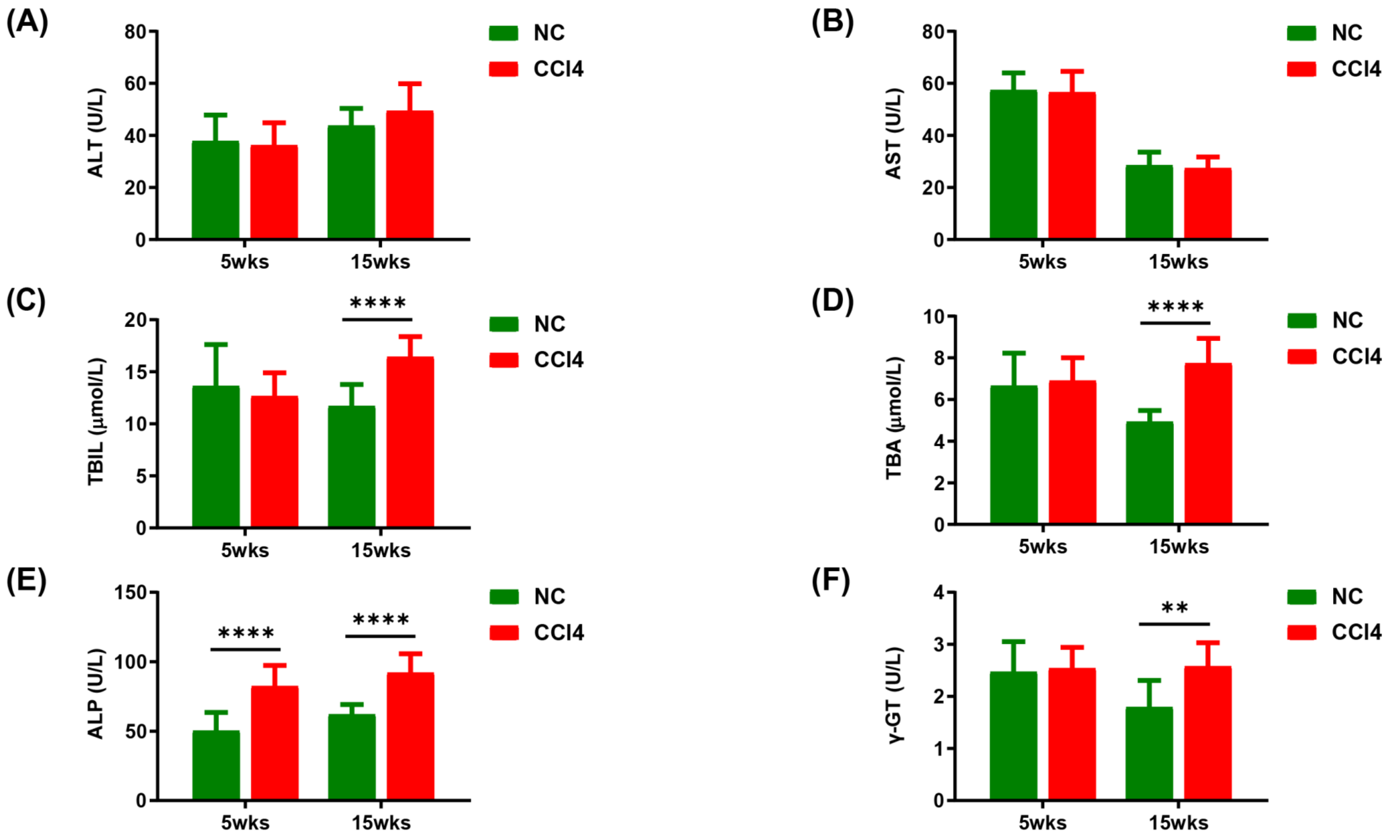

Supplementary Figure 1. CCl_4-induced liver injury in mice. (A-C) The serum indicators of hepatocyte injury including ALT, AST and TBIL, and (D-F) serum parameters of cholangitis and cholestasis including TAB, ALP and γ-GT were measured in mice after 5 or 15-week CCl_4 intervention. Note: Data were given as mean ± SD. n: (A-F) 10 per group. $**P < 0.01$; $****P < 0.0001$. Abbreviations: NC, normal control; CCl_4, carbon tetrachloride; ALT, aminotransferase; AST, aspartate aminotransferase; TBIL, total bilirubin; TBA, total bile acid; ALP, alkaline phosphatase; γ-GT, gamma-glutamyltransferase; SD, standard deviation.

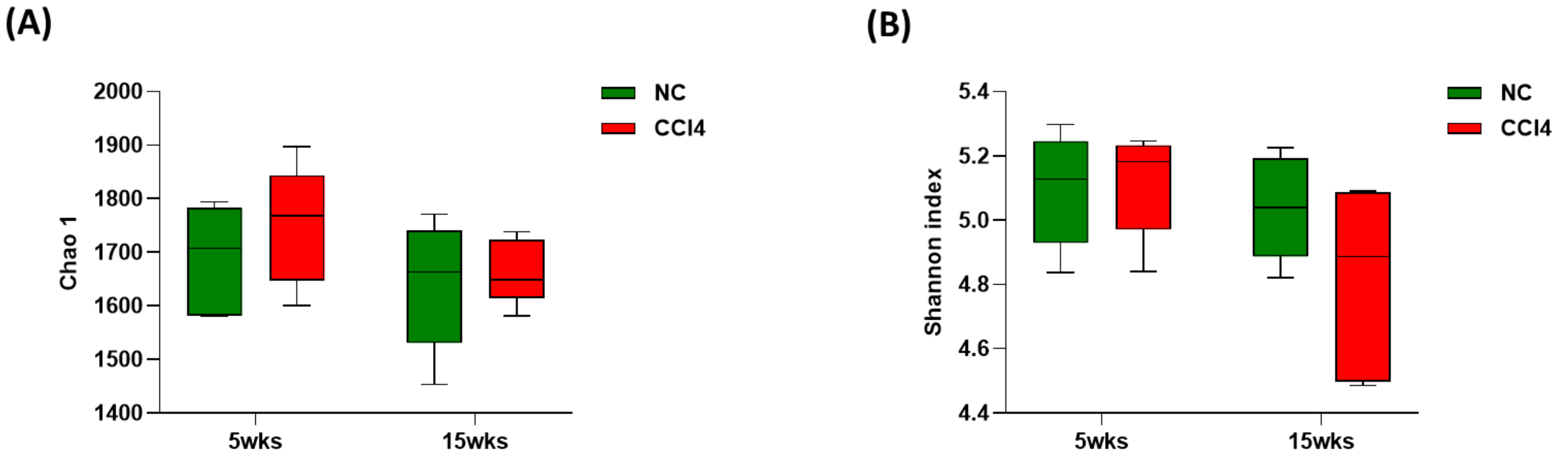

Supplementary Figure 2. The microbial diversity was unchanged in mice during the development of CCl_4-induced liver fibrosis. (A-B) The α-diversity parameters reflecting microbial richness (Chao 1) and diversity (Shannon index) were calculated in mice after 5 or 15-week CCl_4 intervention. Note: Data were given as medians with range. n: (A-B) 5 per group. Abbreviations: NC, normal control; CCl_4, carbon tetrachloride.
